# Improving dental students’ dexterity and strength: an intervention study

**DOI:** 10.1186/s12909-025-08015-8

**Published:** 2025-10-17

**Authors:** María Pérez-Chicharro, María José González-Olmo, Carolina Nieto-Moraleda, Paula Obeso-Benítez, Rosa M Martínez-Piédrola, Patricia Sánchez-Herrera-Baeza, Marta Pérez-de-Heredia-Torres, Martín Romero-Maroto, Elisabet Huertas-Hoyas

**Affiliations:** 1 Department of Nursing and Dentistry, Rey Juan Carlos University, Alcorcón, Spain; 2 Department of Physical Therapy, Occupational Therapy, Rehabilitation, and Physical Medicine, Rey Juan Carlos University, Alcorcón, Spain

**Keywords:** Manipulative dexterity, Psychomotor training, Dental education, Sensory sensitivity, Pre-post study, First-year dental students

## Abstract

**Introduction:**

Dental students require manual dexterity, strength, and sensitivity to execute clinical procedures accurately. However, these skills are often not assessed during the university admission process, despite their relevance for academic and professional performance. The primary objective of this study was to investigate whether a specific home training program improves manual dexterity, strength, and tactile sensitivity in first-year dental students.

**Methods:**

A pre-post intervention study without a control group was carried out with 42 first-year dental students from a Spanish university. Participants followed a home-based programme guided by occupational therapists for eight weeks. Manual dexterity (Nine Hole Peg Test and Box and Block Test), grip strength (dynamometry with JAMAR), and tactile sensitivity (two-point discrimination test) were assessed before and after the intervention. Non-parametric statistical tests were applied, and effect sizes were calculated.

**Results:**

Statistically significant improvements in tactile sensitivity (r >.29, p < .01) and manual dexterity (r >.26, p < .05) in both hands were observed after the intervention. In contrast, grip strength decreased significantly (r >0.35; p < .01). Negative associations were identified between increased sensitivity in the ring and little fingers and reduced strength in both hands. Correlations were also found between improved dexterity and decreased sensitivity in certain fingers of the non-dominant hand. About sex, women showed a greater improvement in sensitivity, although without reaching statistical significance.

**Conclusions:**

A structured home programme significantly improves manual dexterity and sensitivity in dental students, even without expensive equipment, demonstrating that these skills can be effectively trained. However, the decrease in grip strength suggests the desirability of incorporating strengthening exercises in future programmes. It is recommended to include control groups and expand the sample size to confirm these findings and optimise preclinical interventions.

**Supplementary Information:**

The online version contains supplementary material available at 10.1186/s12909-025-08015-8.

## Introduction

Manual dexterity is defined as a person’s ability to manipulate objects with skill and precision with the hands, including components of fine and global motor skills, hand-eye coordination, tactile sensitivity, and graded force control [1, 2]. Dental students require fine manipulative skills for the performance of many of their tasks, as well as a high level of sensitivity, grip strength, and finger strength, e.g., for wire manipulation, cavity preparation, tooth grinding, or manipulation of rotary instruments [3]. Furthermore, even perceptual-spatial skills, such as indirect or mirror vision, are necessary for some of the tasks of dentists [4, 5]. Research shows that, in addition to scientific knowledge, dentistry requires high-level sensory and motor functions, especially in the hands and fingers, on the part of the practitioner in order to carefully and accurately treat the teeth and other organs of the mouth [6–9]. Despite their relevance, manual skills are rarely considered in the admission process to dentistry studies [3, 10], even though these skills will be one of the main pillars for the effective achievement of their tasks [5]. In Spain, there are no specific tests of this type at university entrance; therefore, students entering the first year may present very heterogeneous levels of manipulative skills, although precise handling of instruments will be essential at later stages [11].

Preclinical training tries to compensate for these differences through practice with artificial teeth or mouths, exercises with mirrors for indirect vision, and virtual reality simulators, before practising on patients [3, 12–14]. If students do not reach minimum skills, they will not be able to access and treat patients [15]. Lugassy et al. (2019, conducted an intervention study to improve manual dexterity in dental students. To do so, they used a simulator with indirect vision, which helped to improve these skills in the group of participants compared to the control group. Several studies have conducted preclinical skills training in dental students with virtual reality simulators [14, 16, 17], although the results can be good with the use of this type of technology, the expectations may be higher than the results [18]. In addition, this type of technology can be expensive and conditional on the ownership of the licensing faculty to be able to provide it to their students at set times and with limited use, as opposed to home training, which is convenient and inexpensive.

There is evidence that frequent motor practice, even in non-clinical contexts, such as repetitive manual hobbies, is associated with improved manual dexterity in health science students [19]. However, to the extent of our review, the literature does not describe structured, low-cost, home-based programmes specifically targeted at healthy first-year dental students aimed at optimising their manual dexterity before clinical training. This gap suggests a pedagogical opportunity: to offer early, accessible, and repeatable training that strengthens basic manual components and potentially reduces initial inequalities between students.

Therefore, our hypothesis is based on the fact that after specific home training in manual dexterity, strength, and sensitivity, there will be a significant improvement in these variables. The main objective of the present study was to evaluate the effect of this programme in first-year dental students, comparing pre (T0) and post (T1) intervention measurements. As secondary objectives, the impact of the intervention was analysed according to gender and the correlation between the changes in the different variables.

## Methodology

### Study design

This study used a single-group pre-post design due to ethical and logistical constraints, as the intervention was integrated into a mandatory academic activity. Although the absence of a control group and randomisation limits the internal validity, this pragmatic approach allowed the inclusion of the entire student cohort, enhancing ecological validity in the educational context.

A pre-post intervention study was carried out, which the ethics committee of the Rey Juan Carlos University approved according to the ethical principles for medical research in humans of the Declaration of Helsinki adopted at the 18th Assembly of the World Medical Association (WMA) (Helsinki, Finland, June 1964) and its subsequent revisions. After recruiting the study participants, informed consent was obtained from them for inclusion in the research.

### Sample

After signing the informed consent, 42 volunteer participants were recruited as first-year dental students at the Rey Juan Carlos University, Spain. The sample of participants was obtained by non-probabilistic convenience sampling, although the sample size was calculated a priori. The sample of participants was obtained by non-probabilistic convenience sampling, as all first-year dental students enrolled in the course during the academic year were invited to participate.

The sample size was calculated a priori using G*Power 3.1 for a Wilcoxon signed-rank test, given the study design, establishing a moderate effect size of 0.5 based on Cohen (1988) and previous studies on manual dexterity interventions in health science students, a statistical power of 0.80, and a significance level of 0.05 (two-tailed), resulting in a minimum estimate of 34 participants to detect significant differences; Considering possible losses, 42 students were initially recruited, of whom 37 completed the study, exceeding the minimum required for statistical validity of the results.

The inclusion criteria were the following: being a student of the Degree of Dentistry of the Rey Juan Carlos University; being in the first year of such studies; and being over 18 years of age. The exclusion criteria were: not being enrolled in several courses; having an injury in at least one of the upper limbs; and not signing the informed consent form.

### Assessment instruments

The following assessment instruments were used to measure the students’ manual dexterity. All of them were selected for their psychometric properties, both in terms of validity and reliability, and sensitivity to detect impairments in manual dexterity:

- 2-point discrimination: [20] A Weber-type aesthesiometer was used to measure fine tactile sensitivity at the fingertips of both hands, following the procedure described by Erçalık and Özkurt (2022). This test consists of alternately applying one or two sharp stimuli to the skin, with the participant’s eyes closed, and with light but sufficient pressure to activate the skin receptors without generating pain. The examiner progressively reduces the distance between the two points until the threshold is determined at which the subject can no longer distinguish between one or two contacts. The test was repeated three times per finger, recording the average value. According to Erçalık and Özkurt, reference values in healthy young adults range between 2 and 4 mm, with higher sensitivity in the index and middle fingers, and better results in the dominant hand and women. This test is useful for detecting fine sensory disturbances and for establishing pre-post intervention comparisons in training and clinical contexts.
- Nine-Hole Peg Test (NHPT) [21] is a widely used test to assess fine manual dexterity, especially eye-hand coordination, manipulation speed, and digital accuracy. It is a simple, fast, and highly sensitive tool to change, especially useful in educational and clinical settings. The test involves inserting and then removing nine small pegs from a pegboard with holes, as quickly as possible, using only one hand. It is performed first with the dominant hand and then with the non-dominant hand. The total time required to complete the task is recorded with a stopwatch. Standardised instructions are given, and the participant must be seated, with the forearm resting on a stable surface. According to the study by Temporiti et al. (2022), in healthy young adults (18–30 years), the average time ranges between 16 and 20 s for the dominant hand, and between 17 and 22 s for the non-dominant hand. A shorter time indicates greater fine manual dexterity. In a young, healthy population, a difference of more than 2 s between the two hands could suggest a relevant functional asymmetry. Furthermore, a reduction of at least 1 s in repeated measurements can be considered clinically significant, especially in settings such as pre-clinical dental training. This makes the NHPT a useful tool not only for initial screening, but also for monitoring improvements after specific interventions. This study confirmed that the NHPT has high inter- and intra-rater reliability (ICC >0.90) and that it can capture subtle differences in manual dexterity in a healthy population.
- Box and Block Test: [22] measures unilateral gross manual dexterity by moving small wooden blocks from one compartment to another for 60 s, using only one hand. According to the normative data of Mathiowetz et al. (1985) in healthy adults aged 20–34 years, men typically move 78–85 blocks/minute with the dominant hand and women 71–78 blocks/minute. A difference of up to 10 blocks between the dominant and non-dominant hand is expected. Higher scores indicate greater efficiency in gross motor tasks such as grasping, transferring, and releasing objects. A significant difference between hands or an improvement of 5 blocks or more may be clinically relevant. This test is useful in educational settings to detect difficulties in manipulating dental instruments.
- JAMAR dynamometer [23]: Isometric grip strength was measured using the JAMAR dynamometer, considered the gold standard for this measurement. Participants performed three maximal attempts with each hand, in a seated position, with the elbow flexed at 90° and the forearm at the side of the body; the mean was recorded in kilograms. According to Peters et al. (2011) and the revised values from the PeriNomS study, normative values for grip strength in young adults (20–34 years) are approximately 45–52 kg in men with the dominant hand and 27–32 kg in women with the same dominant hand. Grip strength correlates with overall upper limb function, muscle mass, and the ability to perform clinical tasks requiring sustained pressure. Changes of more than 3–5 kg between assessments can be considered clinically relevant.

These assessments were performed at week 0, before the training programme, and at week 8 after the training programme.

### Intervention

For ethical reasons, as it was not considered appropriate to improve the skills of some students and not others, all were offered the possibility to participate.

All participants were instructed by three occupational therapists (approximately one hour) on how to do the exercises at home and the time to devote to the exercises. The training consisted of five days a week (approximately 20–30 min a day), for 8 weeks. There were three types of tasks to be achieved: daily, weekly, and monthly. All exercises required the performance of both hands. All participants received a booklet explaining the exercises with pictures or videos, with a short text.

All activities had to be recorded by video or photograph and sent by WhatsApp to one of the project researchers. To check adherence to the programme, a message was sent to participants every two weeks to answer any questions.

The designed exercise programme was based on dexterity training programmes [24–27] to achieve key components such as: Fine-tuned control of force, such as in precision grip when picking up small objects; grip strength; gross motor coordination; hand-eye coordination; praxias; surface sensitivity; spatial orientation. A detailed description of the exercises can be found in Appendix 1.

### Statistical analysis

The analysis of the variables was carried out with the statistical programme IBM SPSS Statistics for Windows, version 27.0 (Copyright© 2013 IBM SPSS Corp.).

First, the normality of the sample was tested using the Shapiro-Wilk’s statistic. When this was not confirmed, non-parametric statistics were used. An analysis of the descriptive statistics was performed according to age, sex, and dominance. The Wilcoxon test was used to evaluate the evolution of the variables of sensitivity, strength, and dexterity pre- and post-intervention under the assumption of non-normality. To estimate the differences between groups, the effect size was calculated, and Rosenthal’s r coefficient (r) was used, as these were non-parametric distributions. Interpretation was based on Cohen’s (1988) guidelines for interpreting the results (0.1 = “small effect”, 0.3 = “medium effect”, and 0.5 or higher = “large effect”). To determine whether there are differences in sensitivity, strength, and dexterity after the intervention according to gender, the Mann-Whitney U-test was used. Finally, correlations between variables were analysed according to course and manual dexterity, using Spearman’s non-parametric test. The significant p-statistic value was determined at *p* <.05.

## Results

### Descriptive analysis

The sample consisted of 42 students in the 1 st year of dentistry, who were given a series of tests to assess their manual dexterity, sensitivity, and strength. The measurements were taken at two points in time, before and after the intervention, in order to evaluate the influence of the intervention on the variables measured. Of these 42 records, 5 were eliminated because they did not present results in the post-intervention tests, leaving 37 participants in the study.

The sample was predominantly female, with 83.8% of the sample being female and 16.2% male. In terms of dominance, we see that the participants are mostly right-handed, with only one left-handed person in the whole sample (2.7% of the total). Participants range in age from 18 to 28 years, with the mean age being 19.43 years and the median 18.00 years. Variability is low, with a standard deviation of 2.64 years (Table 1).

**Table 1:** Baseline characteristics of the sample

Category	*N*	%
**Sex**		
* Man*	6	16.2%
* Woman*	31	83.8%
**Dominance**		
* Left-handed*	1	2.7%
* Right-handed*	36	97.3%

### Evolution from T0 to T1 of sensitivity, strength, and dexterity variables

Hypothesis tests were performed to determine whether the differences after intervention in the variables of sensitivity, strength, and dexterity are statistically significant (Table 2).

**Table 2:** Descriptive statistics and results of the pre-and post-intervention hypothesis tests for the variables of sensitivity, strength and dexterity. Test: Wilcoxon test

*Variable*	*Mean (SD) T0*	*Mean (SD) T1*	*Dif. of averages*	*Statistician*	*r*
*Two-point discrimination*					
*Thumb D*	2.59 (0.64)	2.27 (0.51)	−0.32	−2.53*	**0.29**
* Index D*	2.59 (0.60)	2.22 (0.42)	−0.37	−3.13**	**0.36**
* Heart D*	2.92 (0.55)	2.24 (0.43)	−0.68	−4.81**	**0.55**
* Ring D medium D*	2.97 (0.73)	2.3 (0.46)	−0.67	−4.08**	**0.47**
*Annular ulnar D*	2.86 (0.75)	2.27 (0.45)	−0.59	−3.74**	**0.43**
* Littlefinger D*	2.92 (0.76)	2.22 (0.42)	−0.70	−4.29**	**0.49**
* Thumb ND*	2.54 (0.51)	2.24 (0.43)	−0.30	−2.52*	**0.29**
*Index ND*	2.59 (0.60)	2.24 (0.43)	−0.35	−2.71**	**0.31**
*Heart ND*	2.68 (0.58)	2.35 (0.48)	−0.33	−2.68**	**0.31**
*Annular medium ND*	2.78 (0.63)	2.43 (0.50)	−0.35	−2.84**	**0.33**
* Annular ulnar ND*	2.81 (0.70)	2.43 (0.50)	−0.38	−2.99**	**0.34**
* Pinky ND*	2.84 (0.80)	2.32 (0.53)	−0.52	−2.93**	**0.34**
*Dynamometer*					
* Dynamometer D*	30.01 (7.82)	27.68 (7.16)	−2.33	−3.02**	**0.35**
* ND Dynamometer*	28.36 (8.75)	25.51 (8.53)	−2.85	−3.80**	**0.44**
*Nine hole*					
* Nine -Hole D*	18.42 (2.17)	17.25 (2.33)	−1.17	−3.03**	**0.35**
* Nine-Hole ND*	19.94 (2.40)	18.7 (2.57)	−1.24	−2.44*	**0.28**
*Box & Block*					
* Box & block D*	59.11 (10.51)	64.59 (9.44)	5.48	−2.46*	**0.28**
* Box & block ND*	58.92 (10.13)	61.78 (8.61)	2.86	−2.24*	**0.26**

*D* Dominant, *ND* Non-dominant *r* Rosenthal’s R

* Significant (*p* <.05) and ** Highly significant (*p* <.01)

After the intervention, there is an overall improvement in sensitivity, as in all two-point discrimination tests, there are significant differences with a small effect size (*r* >.29, p <.

0.01). Similarly, strength has significantly decreased with a medium effect size (*r* >.35, *p* <.01), as we obtain positive results in all dynamometer tests. In addition, we found a significant improvement in dexterity with a small effect size (*r* >.26, *p* <.05) in the Nine Hole for both hands, and in B&B, we see that both the dominant and non-dominant hands showed significant increases.

### Sex differences

In order to determine whether the evolution of the participants depends on gender, differences between time points were calculated for each variable and participant. The description of these changes is shown separately for each sex. Looking at the descriptive, we see that the changes are similar in both groups. We apply a hypothesis test comparing the changes in each of the variables between men and women.

The results indicate significant differences in the two-point discrimination tests of the thumb ND (Male 0.33 ± 0.52, Female − 0.42 ± 0.62; *p* <.05), middle ND (Male 0.33 ± 0.52, Female − 0.45 ± 0.62; *p* <.05), and middle ring ND (Male 0.33 ± 0.52, Female − 0.48 ± 0.63; *p* <.05). In all these cases, we observe that the changes in females have a negative mean, while in males, they are positive. This would indicate that females have improved in discrimination tests, while males have worsened. These results should be considered exploratory and should be interpreted with caution. In the absence of a larger sample size for men, we can conclude that there are indications of a differential evolution of sensitivity during the intervention. Sensitivity seems to improve in women, but worsens in men.

### Relationship between the variables under study

To determine how the results of the different tests co-evolved, a correlation analysis was carried out, correlating the T0-T1 differences (Table 3). An increase in the sensitivity of the medial ring finger (r2 = 0.396*) and ulnar ring finger (r2 = 0.394*) in the dominant hand is associated with a loss of strength in the dominant hand, while in the non-dominant hand, the association is in the same direction, but in the meniscus (r2 = 0.632**). In addition, an increase in dexterity is associated with a decrease in sensitivity in the medial annulus (r2 = 0.416*), ulnar annulus (r2 = 0.328*), and cardia (r2 = 0.356*) of the non-dominant hand.

**Table 3:** Non-parametric correlation matrix (Spearman’s Rho). Time: differences between pre-intervention and post-intervention

T0-T1	1	2	3	4	5	6	7	8	9	10	11
Ring medium D											
Annular ulnar D	0.740**										
*Heart ND*	0.197	0.240									
*Override ND*	0.221	0.306	0.509**								
*Annular ulnar ND*	0.273	0.293	0.189	0.592**							
*Pinky ND*	0.351*	0.338*	0.185	0.272	0.632**						
*Dynamometer D*	− 0.396*	− 0.394*	− 0.010	0.032	− 0.125	− 0.037					
*ND Dynamometer*	− 0.448**	− 0.356*	− 0.010	− 0.027	− 0.255	− 0.244	0.816**				
*Nine-Hole D*	− 0.180	− 0.096	− 0.345*	− 0.032	− 0.399*	− 0.229	− 0.006	0.170			
*Nine-Hole ND*	− 0.243	− 0.207	− 0.008	− 0.416*	− 0.328*	− 0.183	− 0.062	− 0.080	0.266		
*Box & block D*	0.096	0.065	0.356*	0.154	0.141	0.151	0.053	0.114	0.083	− 0.115	
*Box & block ND*	− 0.063	− 0.015	0.334*	0.066	0.123	0.210	0.054	0.094	0.134	− 0.074	0.721**

*D *Dominant*, ND *Non-dominant

* Significant (*p* <.05) and** Highly significant (*p* <.01)

## Discussion

The present study aimed to test whether a specific training programme in dental students improves manual dexterity, strength, and sensitivity of the upper limbs. As secondary objectives, we explored the impact of the intervention as a function of gender and the relationship between the changes in the different variables evaluated.

In this research, we evaluated dexterity after a specific training programme, obtaining significantly better values in dexterity when comparing the scores obtained in pre- and post-training. These results are consistent with previous literature, as in studies by [3, 7, 10, 12, 28–30], which concluded that manual dexterity can be acquired and improved through exercise.

Manual dexterity requirements for dental practice are unknown; manual dexterity, stereopsis, hand-eye coordination, cognitive, perceptual, handwriting, and drawing skills in dentistry are recurrent research topics [31–33]. Therefore, in our study, we took into account two more variables, such as sensitivity and strength.

About sensitivity, the results we obtained in this study were a significant improvement after the training programme, but no research has evaluated the difference in two-point discrimination after an intervention in healthy subjects. We do find numerous studies [20, 34–37] descriptive that assess sensitivity in young adults aged 18–28 years, as we found in this study, in different areas of the upper extremity: arm, forearm, palmar surface of the hand and palmar surface of the distal and middle phalanges of the finger, thumb, middle and little finger. The results we obtain in this analysis are consistent with other studies [20, 34–37], in terms of higher sensitivity found in the fingertips. It is in these areas that the two points can be discriminated over a smaller distance compared to other parts of the body. This may be due to the higher density of receptors and free nerve endings in the distal areas, resulting in greater sensitivity [20, 34].

Therefore, this finding provides original evidence for possible sensory plasticity in students without pathology.

On the other hand, strength is another variable that we measured pre- and post-intervention, where we found a significant decrease after the intervention, an unexpected finding considering that most physical intervention programmes tend to maintain or improve this variable. This may be due to the fact that the training emphasized fine motor control and bilateral coordination rather than muscle strengthening. Thus, participants may have experienced neuromotor adaptations prioritizing precision over maximal grip force. It is also possible that repetitive use of the muscles without rest led to transient fatigue, affecting grip measurements. As with sensitivity, we found no articles assessing strength in healthy subjects after intervention. Chen CY et al., 2018 [38] assesses intrinsic hand muscle strength in young people aged 13–20 years and determines that strength can be conditioned by numerous factors such as gain or loss of muscle volume, change in body composition, the different occupations of each participant and fine motor development; therefore, as these variables were not taken into account, we cannot determine the cause of the decrease in strength after the intervention. In our case, the training was oriented towards bilateral precision and fine coordination activities, with little or no sustained strength work involved. This could have favoured a neuromotor adaptation oriented towards precision and coordination, to the detriment of global maximal strength.

About gender, and taking into account the small sample of men available to us, we found a weak correlation between gender and the level of improvement, as both sexes improved significantly in terms of dexterity, as supported by the study conducted by Saeed et al., 2022 [28]. In contrast, Lugassy et al., 2019 [12], showed that females performed somewhat better than males in activities requiring fine motor skills, in line with the findings of [39].

With regard to sensitivity in studies conducted by different authors [20, 34, 35], there were no significant differences between the two sexes. In contrast [36, 37, 40], found that women have higher sensitivity in upper limbs than men, which may improve the reliability of the present study, since in the results, we found that women improved sensitivity after the intervention and therefore obtained higher values than men. Although this trend exists, the difference between both sexes is not statistically significant, at the expense of increasing the sample of men. This could be attributed to the fact that the area of the sensory homunculus corresponds to the relative size of the receptive field in the cerebral cortex. The female sex has larger receptive fields of the cortex controlling the hand and mouth senses [36].

Finally, about strength, both Chen et al., 2017 [38] and Fietsam et al., 2022 [41] argue that adolescent and young adult males possess greater intrinsic hand muscle strength than females, although it should be noted that measuring grip strength using dynamometry can be affected by hand size. In addition, this grip strength correlates with overall strength and body composition. These data are likely to be influenced by the low sample size of men.

The results of this study show that as sensitivity increases, strength decreases, especially in the fingers of the dominant hand. No studies have been identified that explicitly document this relationship in a young healthy population. Olafsdottir HB et al., 2008 [42] show in their research that strength training improves more in the proximal than in the distal part of the fingers. This could suggest that an increase in proximal strength may lead to improvements in sensitivity and dexterity in the distal areas, as a result of a readjustment in neuromuscular control. This would suggest that increased strength in the proximal zones of the upper limb may facilitate improvements in sensitivity and dexterity in the distal zones as a result of a readjustment in neuromuscular control. This process would involve greater activation of small, precise motor units, rather than a predominant use of brute force, which would support the results observed in this study.

This finding opens a novel line of research on the interaction between sensitivity and force in clinical tasks with high manipulative demands, such as those in the dental field.

The present research has several limitations. Firstly, the sample size, being a pilot study, the sample group was limited to 1 st year dental students from our faculty, which directly affects the external validity of the results and limits their generalisability to other dental student populations. As the sample consisted of 42 students (with complete data from 37 participants) from a single university cohort, the results should be interpreted with caution. On the other hand, gender was unbalanced due to the large number of women studying dentistry [43]. Another limitation is that we did not take into account whether the study participants had any hobbies or hobbies that required high manual dexterity, such as embroidery or drawing, or whether they practised any sport that affected the variables measured. We also did not take into account whether they had any other previous training before entering university related to the dental field, such as dental prosthetics or dental hygiene. However, we tried to make the sample homogeneous in terms of age and maturational development. Therefore, further research with a larger sample size is needed to obtain more reliable results. In addition, the lack of a control group, randomisation, and prior familiarisation with the evaluation tools introduces bias and limits the ability to attribute observed changes solely to the intervention. These limitations affect internal validity and reduce reproducibility. Future studies should incorporate controlled designs and repeated measurements over time to increase robustness.

Nevertheless, this study has important clinical implications. The results obtained in this study show that providing a specific, accessible, and low-cost training programme improves the manual dexterity and sensitivity of dental students, without having to resort to other programmes with significantly higher costs, such as virtual reality [14, 16, 17] or the development of a portable phantom [12]. The programmed intervention included simple explanations and pictures, which made the programme very comprehensible. In addition, weekly training reports from most subjects suggested high adherence.

Application in different dental schools may benefit the learning process of students in the preclinical phase.

Possible lines of research could be directed towards comparison with a control group not receiving the specific complementary intervention in order to observe the effect of the programme in the long term and at different educational stages.

## Conclusions

- After the implementation of an intervention programme in dental students, a significant improvement in manual dexterity and sensitivity was observed, as well as a significant decrease in strength.
- About sex, a significant improvement in dexterity was observed in both groups, with no significant differences between men and women; however, in terms of sensitivity, we did find a greater improvement in women, although this was not statistically significant. Regarding strength, a decrease was observed in both sexes.
- Finally, an inverse association was found between sensitivity and strength, suggesting that the increase in sensitivity could be related to a lower grip strength. This finding suggests a possible functional interaction between the two variables that should be explored in future studies. 

## Supplementary Information

Supplementary Material 1.

## Data Availability

No datasets were generated or analysed during the current study.

## Abbreviations

NHPT: Nine-Hole Peg Test
B&B: Box and Block Test
ICC: Intraclass Correlation Coefficient
WMA: World Medical Association
T0: Time before the intervention
T1: Time after the intervention
r: Rosenthal’s effect size coefficient
Mm: Millimeters
D: Dominant
ND: Non dominant
Kg: Kilograms

## References

[1] Vasylenko O, Gorecka MM, Rodríguez-Aranda C. Manual dexterity in young and healthy older adults. 1. Age- and gender-related differences in unimanual and bimanual performance. Dev Psychobiol. 2018;60(4):407–27. 10.1002/dev.21619. 29528105 10.1002/dev.21619

[2] Wilson N, Hough E, Hamilton A, Verdonck M, Clark R. Development and test-retest reliability assessment of a low-cost, 3D printed tool for assessing different aspects of hand dexterity. J Hand Ther. 2023;36(1):133–8. 10.1016/j.jht.2021.06.005. 34304977 10.1016/j.jht.2021.06.005

[3] Lugassy D, Levanon Y, Pilo R, Shelly A, Rosen G, Meirowitz A, Brosh T. Predicting the clinical performance of dental students with a manual dexterity test. PLoS ONE. 2018;13(3):e0193980. 10.1371/journal.pone.0193980. 29518127 10.1371/journal.pone.0193980 PMC5843268

[4] Işik Eİ, Soygun K, Kahraman ÖC, Koçak EF. The effect of the menstrual cycle on the sense of touch, grip strength and manual dexterity of dental students. Int J Occup Saf Ergon. 2022;28(2):1167–75. 10.1080/10803548.2021.1880714. 33482712 10.1080/10803548.2021.1880714

[5] Schwibbe A, Kothe C, Hampe W, Konradt U. Acquisition of dental skills in preclinical technique courses: influence of Spatial and manual abilities. Adv Health Sci Educ Theory Pract. 2016;21(4):841–57. 10.1007/s10459-016-9670-0. 26891678 10.1007/s10459-016-9670-0

[6] Levanon Y, Lugassy D, Pilo R, Nassar R, Mhana A, Maria Z, Willy N, Brosh T. Assessment of the modified o’connor tweezer dexterity and Purdue pegboard test for use among dental students. J Dent Educ. 2023;87(4):533–9. 10.1002/jdd.13137. 36374560 10.1002/jdd.13137

[7] Neves TDC, Pazos JM, Genaro LE, Hallak JC, Garcia PPNS. Manual dexterity in dentistry: development and evaluation of a preclinical training program. J Dent Educ. 2023;87(9):1242–9. 10.1002/jdd.13233. 37160672 10.1002/jdd.13233

[8] Lundergan WP, Soderstrom EJ, Chambers DW. Tweezer dexterity aptitude of dental students. J Dent Educ. 2007;71(8):1090–7. 17687091

[9] de Andrés AG, Sánchez E, Hidalgo JJ, Díaz MJ. Appraisal of psychomotor skills of dental students at university complutense of Madrid. Eur J Dent Educ. 2004;8(1):24–30. 10.1111/j.1600-0579.2004.00296.x. 14717687 10.1111/j.1600-0579.2004.00296.x

[10] Giuliani M, Lajolo C, Clemente L, Querqui A, Viotti R, Boari A, Miani CM. Is manual dexterity essential in the selection of dental students? Br Dent J. 2007;203(3):149–55. 10.1038/bdj.2007.688. 17694029 10.1038/bdj.2007.688

[11] Siman B, Janszky J, Perlaki G, Fazekas A, Sandor B, Katona K, Marada G, Szanto I. Course induced dexterity development and cerebellar grey matter growth of dentistry students: a randomised trial. Sci Rep. 2021;11(1):6188. 10.1038/s41598-021-84549-3. 33731734 10.1038/s41598-021-84549-3 PMC7969763

[12] Lugassy D, Levanon Y, Shpack N, Levartovsky S, Pilo R, Brosh T. An interventional study for improving the manual dexterity of dentistry students. PLoS ONE. 2019;14(2):e0211639. 10.1371/journal.pone.0211639. 30707724 10.1371/journal.pone.0211639 PMC6358065

[13] Rau GM, Rau AK. Training device for dental students to practice mirror-inverted movements. J Dent Educ. 2011;75(9):1280–4. 21890859

[14] Gal GB, Weiss EI, Gafni N, Ziv A. Preliminary assessment of faculty and student perception of a haptic virtual reality simulator for training dental manual dexterity. J Dent Educ. 2011;75(4):496–504. 21460270

[15] Gray SA, Deem LP. Predicting student performance in preclinical technique courses using the theory of ability determinants of skilled performance. J Dent Educ. 2002;66(6):721–7. 12117093

[16] LeBlanc VR, Urbankova A, Hadavi F, Lichtenthal RM. A preliminary study in using virtual reality to train dental students. J Dent Educ. 2004;68(3):378–83. 15038639

[17] Buchanan JA. Experience with virtual reality-based technology in teaching restorative dental procedures. J Dent Educ. 2004;68(12):1258–65. 15576814

[18] Gottlieb R, Lanning SK, Gunsolley JC, Buchanan JA. Faculty impressions of dental students’ performance with and without virtual reality simulation. J Dent Educ. 2011;75(11):1443–51. 22058393

[19] Kuzgun H, Denat Y. The manual dexterity of nursing students and factors that affect it. Int J Occup Saf Ergon. 2020;26(1):9–14. 10.1080/10803548.2018.1442909. 29521574 10.1080/10803548.2018.1442909

[20] Erçalık C, Özkurt S. Two-point discrimination assessment of the upper extremities of healthy young Turkish individuals. Turk J Phys Med Rehabil. 2022;68(1):136–41. 10.5606/tftrd.2022.6263. 35949974 10.5606/tftrd.2022.6263 PMC9305636

[21] Temporiti F, Mandaresu S, Calcagno A, Coelli S, Bianchi AM, Gatti R, Galli M. Kinematic evaluation and reliability assessment of the nine hole Peg test for manual dexterity. J Hand Ther. 2023;36(3):560–7. 10.1016/j.jht.2022.01.007. 35232627 10.1016/j.jht.2022.01.007

[22] Mathiowetz V, Volland G, Kashman N, Weber K. Adult norms for the box and block test of manual dexterity. Am J Occup Ther. 1985;39(6):386–91. 10.5014/ajot.39.6.386. 3160243 10.5014/ajot.39.6.386

[23] Peters MJ, van Nes SI, Vanhoutte EK, Bakkers M, van Doorn PA, Merkies IS, Faber CG, PeriNomS Study group. Revised normative values for grip strength with the Jamar dynamometer. J Peripher Nerv Syst. 2011;16(1):47–50. 10.1111/j.1529-8027.2011.00318.x. 21504502 10.1111/j.1529-8027.2011.00318.x

[24] Pérez-Mármol JM, García-Ríos MC, Ortega-Valdivieso MA, Cano-Deltell EE, Peralta-Ramírez MI, Ickmans K, Aguilar-Ferrándiz ME. Effectiveness of a fine motor skills rehabilitation program on upper limb disability, manual dexterity, pinch strength, range of fingers motion, performance in activities of daily living, functional independency, and general self-efficacy in hand osteoarthritis: A randomized clinical trial. J Hand Ther. 2017;30(3):262–73. 10.1016/j.jht.2016.12.001. 28502698 10.1016/j.jht.2016.12.001

[25] Vanbellingen T, Nyffeler T, Nigg J, Janssens J, Hoppe J, Nef T, Müri RM, van Wegen EEH, Kwakkel G, Bohlhalter S. Home based training for dexterity in parkinson’s disease: A randomized controlled trial. Parkinsonism Relat Disord. 2017;41:92–8. 10.1016/j.parkreldis.2017.05.021. 28578819 10.1016/j.parkreldis.2017.05.021

[26] Budini F, Lowery MM, Hutchinson M, Bradley D, Conroy L, De Vito G. Dexterity training improves manual precision in patients affected by essential tremor. Arch Phys Med Rehabil. 2014;95(4):705–10. 10.1016/j.apmr.2013.11.002. 24275064 10.1016/j.apmr.2013.11.002

[27] Kamm CP, Mattle HP, Müri RM, Heldner MR, Blatter V, Bartlome S, Lüthy J, Imboden D, Pedrazzini G, Bohlhalter S, Hilfiker R, Vanbellingen T. Home-based training to improve manual dexterity in patients with multiple sclerosis: A randomized controlled trial. Mult Scler. 2015;21(12):1546–56. 10.1177/1352458514565959. 25623246 10.1177/1352458514565959

[28] Saeed M, Alfarra MBQ, Abdelmagied MH, Hadi K, Aljafarawi T, Al-Rawi N, Uthman AT, Fanas SA, Al Rawi NH. Comparative analysis of manual dexterity of dental students at Ajman university following one academic year of preclinical training sessions: A longitudinal cohort study. Eur J Dent. 2023;17(4):1179–88. 10.1055/s-0042-1758793. 36535660 10.1055/s-0042-1758793 PMC10756829

[29] Luck O, Reitemeier B, Scheuch K. Testing of fine motor skills in dental students. Eur J Dent Educ. 2000;4(1):10–4. 10.1034/j.1600-0579.2000.040103.x. 11168460 10.1034/j.1600-0579.2000.040103.x

[30] Gansky SA, Pritchard H, Kahl E, Mendoza D, Bird W, Miller AJ, Graham D. Reliability and validity of a manual dexterity test to predict preclinical grades. J Dent Educ. 2004;68(9):985–94. 15342660

[31] Hegarty M, Keehner M, Khooshabeh P, Montello DR. How Spatial abilities enhance, and are enhanced by, dental education. Learn Individ Differ. 2009;19:61–70.

[32] Dimitrijevic T, Kahler B, Evans G, Collins M, Moule A. Depth and distance perception of dentists and dental students. Oper Dent. 2011;36(5):467–77. 10.2341/10-290-L. 21859316 10.2341/10-290-L

[33] Suksudaj N, Townsend GC, Kaidonis J, Lekkas D, Winning TA. Acquiring psychomotor skills in operative dentistry: do innate ability and motivation matter? Eur J Dent Educ. 2012;16(1):e187–94. 10.1111/j.1600-0579.2011.00696.x. 22251344 10.1111/j.1600-0579.2011.00696.x

[34] Shibin K, Asir JS. The discrimination of two-point touch sense for the upper extremity in Indian adults. Int J Health Rehabil Sci. 2013;2(1):38–43.

[35] Alsaeed S, Alhomid T, Zakaria HM, Alwhaibi RM. Normative values of Two-Point discrimination test among students of Princess Noura Bint Abdulrahman university in Riyadh. Int J Adv Phys Al Sci. 2014;1:42–52.

[36] Koo JP, Kim SH, An HJ, Moon OG, Choi JH, Yun YD, Park JH, Min KO. Two-point discrimination of the upper extremities of healthy Koreans in their 20’s. J Phys Ther Sci. 2016;28(3):870–4. 10.1589/jpts.28.870. 27134375 10.1589/jpts.28.870 PMC4842456

[37] Nolan MF. Two-point discrimination assessment in the upper limb in young adult men and women. Phys Ther. 1982;62(7):965–9. 10.1093/ptj/62.7.965. 7089059 10.1093/ptj/62.7.965

[38] Chen CY, McGee CW, Rich TL, Prudente CN, Gillick BT. Reference values of intrinsic muscle strength of the hand of adolescents and young adults. J Hand Ther. 2018;31(3):348–56. 10.1016/j.jht.2017.05.012. 28807597 10.1016/j.jht.2017.05.012 PMC5955806

[39] Upadhayay N, Guragain S. Comparison of cognitive functions between male and female medical students: a pilot study. J Clin Diagn Res. 2014;8(6):BC12–5. 10.7860/JCDR/2014/7490.4449. 25120970 10.7860/JCDR/2014/7490.4449 PMC4129348

[40] Louis DS, Greene TL, Jacobson KE, Rasmussen C, Kolowich P, Goldstein SA. Evaluation of normal values for stationary and moving two-point discrimination in the hand. J Hand Surg Am. 1984;9(4):552–5. 10.1016/s0363-5023(84)80109-4. 10.1016/s0363-5023(84)80109-4 6747241

[41] Fietsam AC, Tucker JR, Kamath MS, Huang-Pollock C, Wang Z, Neely KA. Manual dexterity and strength and in young adults with and without Attention-Deficit/Hyperactivity disorder (ADHD). Neurosci Lett. 2022;766:136349. 10.1016/j.neulet.2021.136349. 34785312 10.1016/j.neulet.2021.136349 PMC9578534

[42] Olafsdottir HB, Zatsiorsky VM, Latash ML. The effects of strength training on finger strength and hand dexterity in healthy elderly individuals. J Appl Physiol. 2008;105(4):1166–78. 10.1152/japplphysiol.00054.2008. 18687981 10.1152/japplphysiol.00054.2008 PMC2576040

[43] Hernández-Ruiz RE, Rosel-Gallardo EM, Cifuentes-Jiménez C, González-López S, Bolaños-Carmona MV. Gender and leadership positions in Spanish dentistry. Inquiry. 2022;59:469580221109970. 10.1177/00469580221109970. 35912432 10.1177/00469580221109970 PMC9340893

