# Supplementary material for: Improving dental students’ dexterity and strength: an intervention study
Source: BMC Med Educ. 2025 Oct 17;25:1436. doi: 10.1186/s12909-025-08015-8 (PMC12532813; doi:10.1186/s12909-025-08015-8)
Supplement: Supplementary file 1 — Supplementary Material 1. [file 12909_2025_8015_MOESM1_ESM.docx]

**Annex: Intervention Programme for the Development of Manipulative Dexterity in Dental Students**

# Objective of the intervention

The main objective of the intervention programme was to improve fine and global manipulative dexterity in dental students through planned activities that integrate precision, bilateral coordination, manual strength and spatial orientation. The tasks were designed to be performed autonomously by the participants, under weekly monitoring and structured feedback.

**Programme Structure**

The programme was developed over a period of 8 weeks, with activities structured at three levels of frequency: daily, weekly and monthly. Each was selected for its ability to stimulate neuromuscular components associated with manual dexterity.

**Monitoring and Evaluation**

Students submitted weekly evidence (photographic or video) as part of programme monitoring.

Adherence was monitored by occupational therapists who conducted introductory sessions and provided support during the intervention.

Pre- and post-intervention measurements were taken at week 0 and week 8 to assess changes in sensation, strength and manipulative dexterity, using psychometrically validated instruments.

# Daily or Fixed Activities

## Mirror writing

The participant must reproduce simple geometric silhouettes (square, star, labyrinth, etc.) looking exclusively through a mirror, without direct vision of the paper.

This makes it possible to work on visual-motor coordination under conditions of spatial inversion. It is done with both hands separately, recording the execution time.

Materials: pen, mirror, sheet of paper, folder or cardboard to hide direct vision.


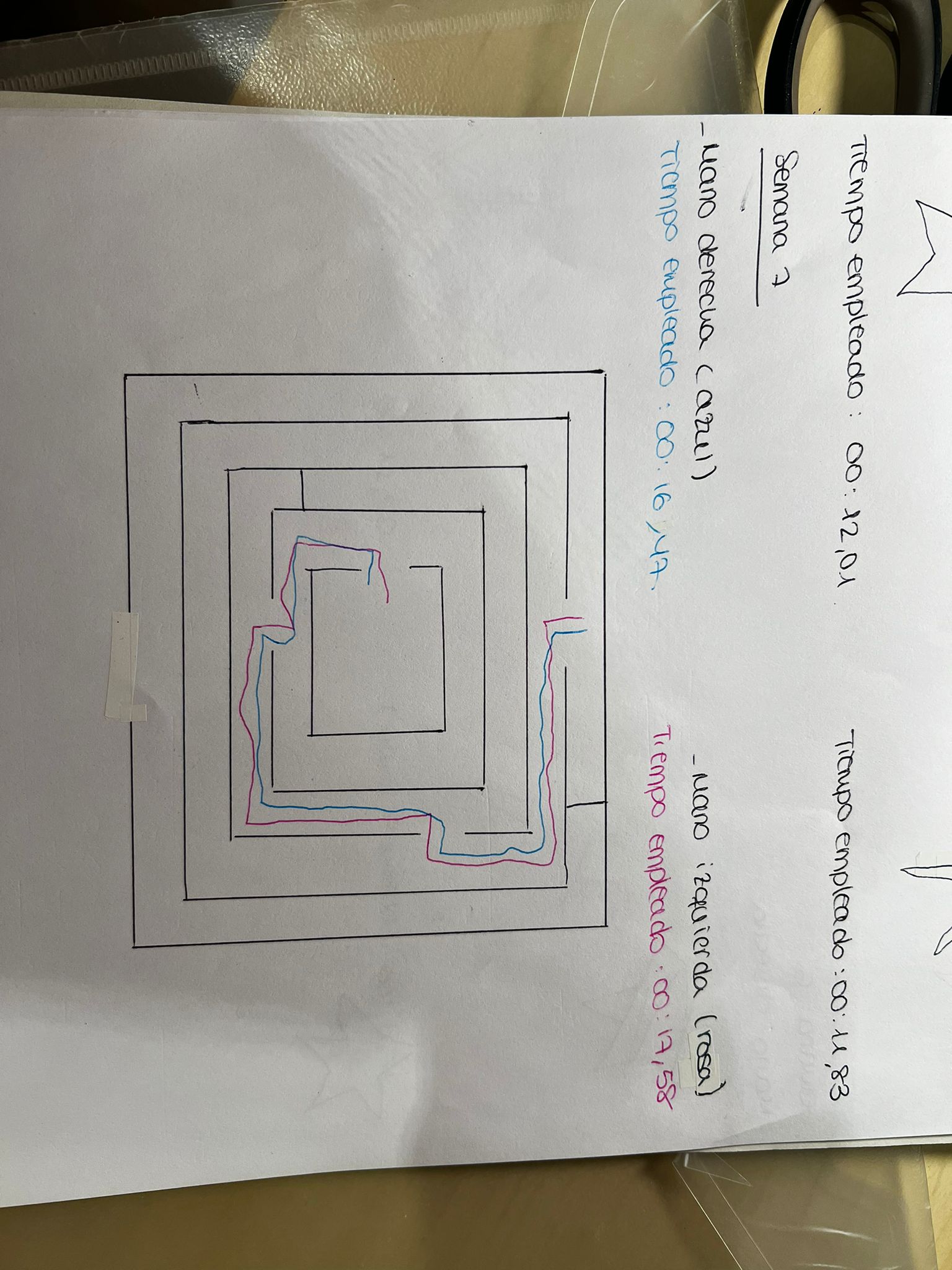


**Bilateral stapling**

The exercise consists of stapling the edges of a set of 12 sheets of paper suspended in the air. The upper and right edge is worked with the right hand and the lower and left edge with the left hand.

This activity strengthens the muscles of both hands.

Materials: sheets of paper, manual stapler, staples.


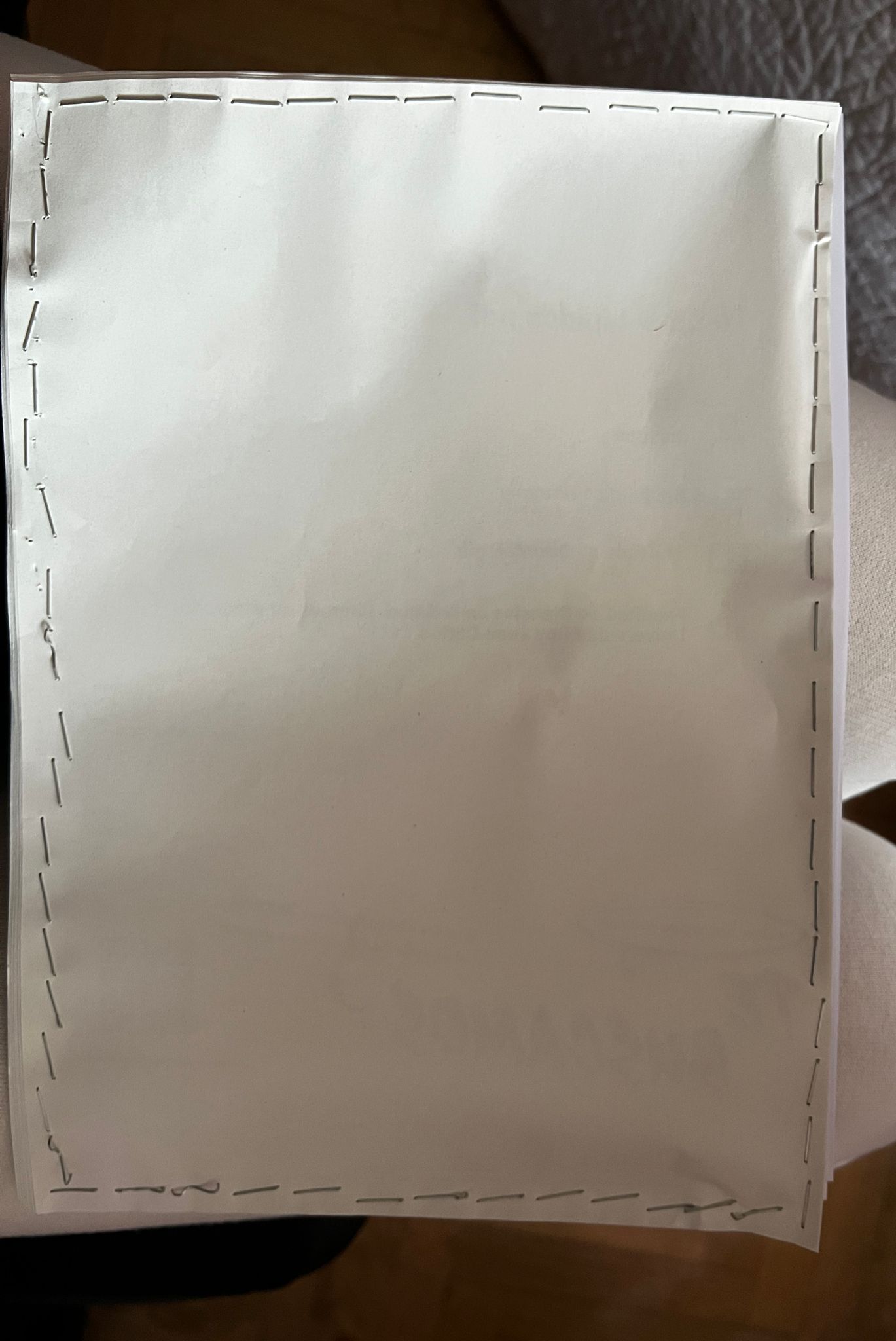


**Making knots**

Each week a new knot is learnt. The student must tie two of the same type of knot. The order and technique of each knot follows a standardised tutorial.

This activity promotes digital dexterity, motor precision and sustained attention.

Materials: macramé thread or rope.


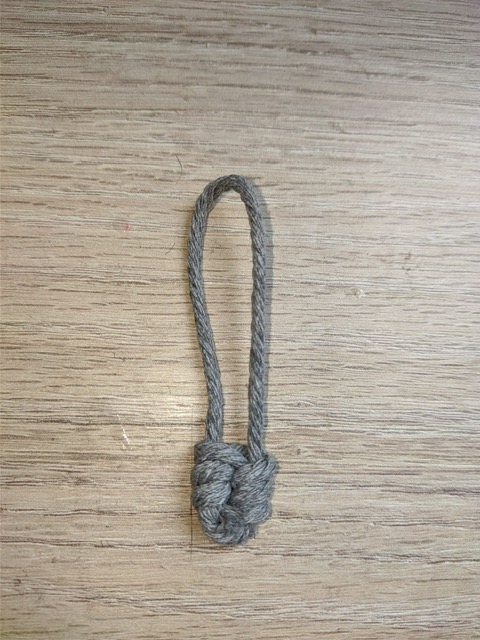


**Clip decoration**

This involves lining metal clips with coloured thread to form decorative figures, especially in the shape of a heart. Two paper clips are made per week.

Stimulates fine motor skills and creativity, as well as repetitive work with small elements.

Materials: paper clips, decorative thread, scissors.


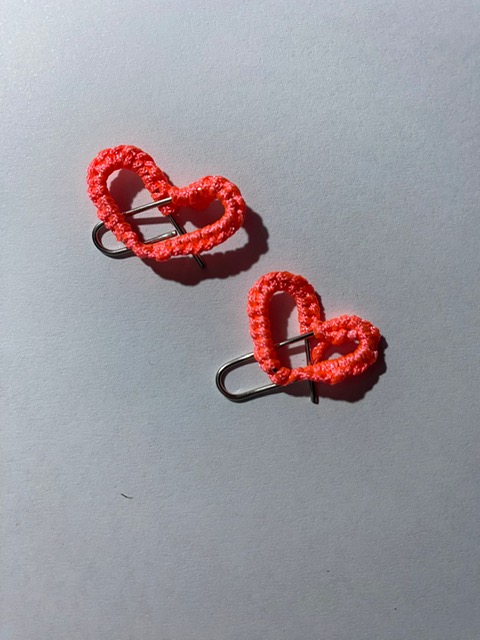


**Star construction with staples**

Staples are grouped in sets of three without separating, forming 12 units, and then assembled into a star shape.

The activity trains precision and hand-eye coordination, as well as structural dexterity.

Materials: loose staples in blocks, flat work surface.


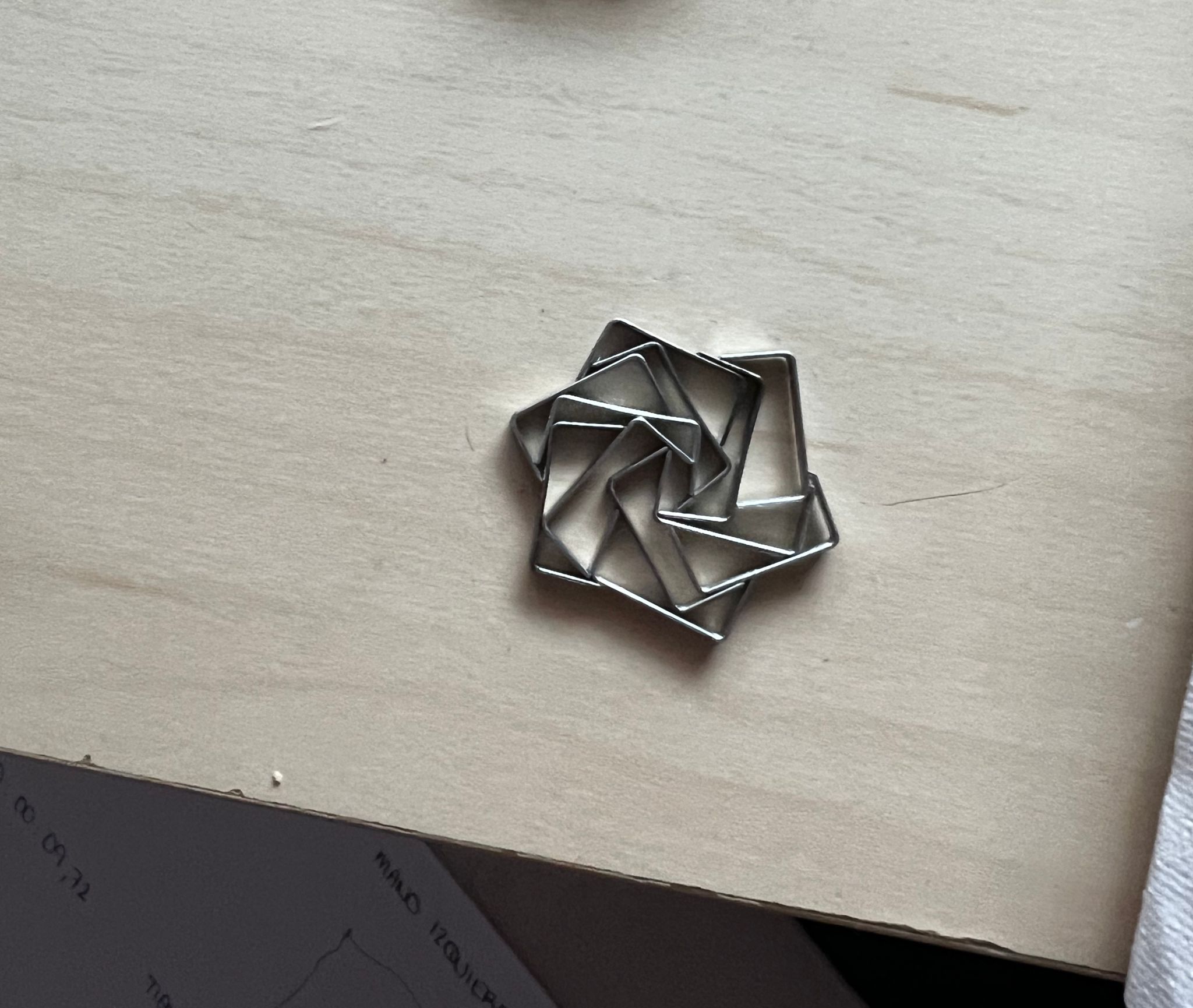


**Weekly activities**

**Drawing with balls of paper**

Consists of creating an artistic design using balls of tissue paper, which are attached with white glue to a cardboard base with the help of tweezers.

They work on fine tweezers, bimanual coordination and orientation in graphic space. Materials: tissue paper, white glue, fine tweezers, cardboard.


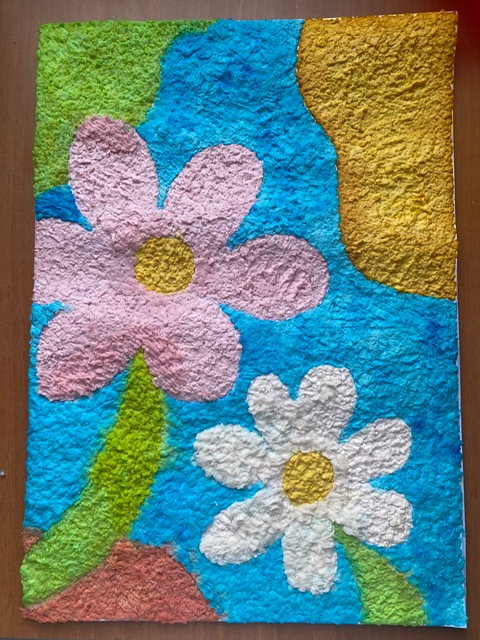


**Model aeroplane construction**

A model aeroplane is constructed using tongue depressors, plasticine and glue. Students follow a logical sequence of assembling parts.

This activity encourages motor planning and constructive motor skills.

Materials: wooden tongue depressors, scissors, glue, plasticine.


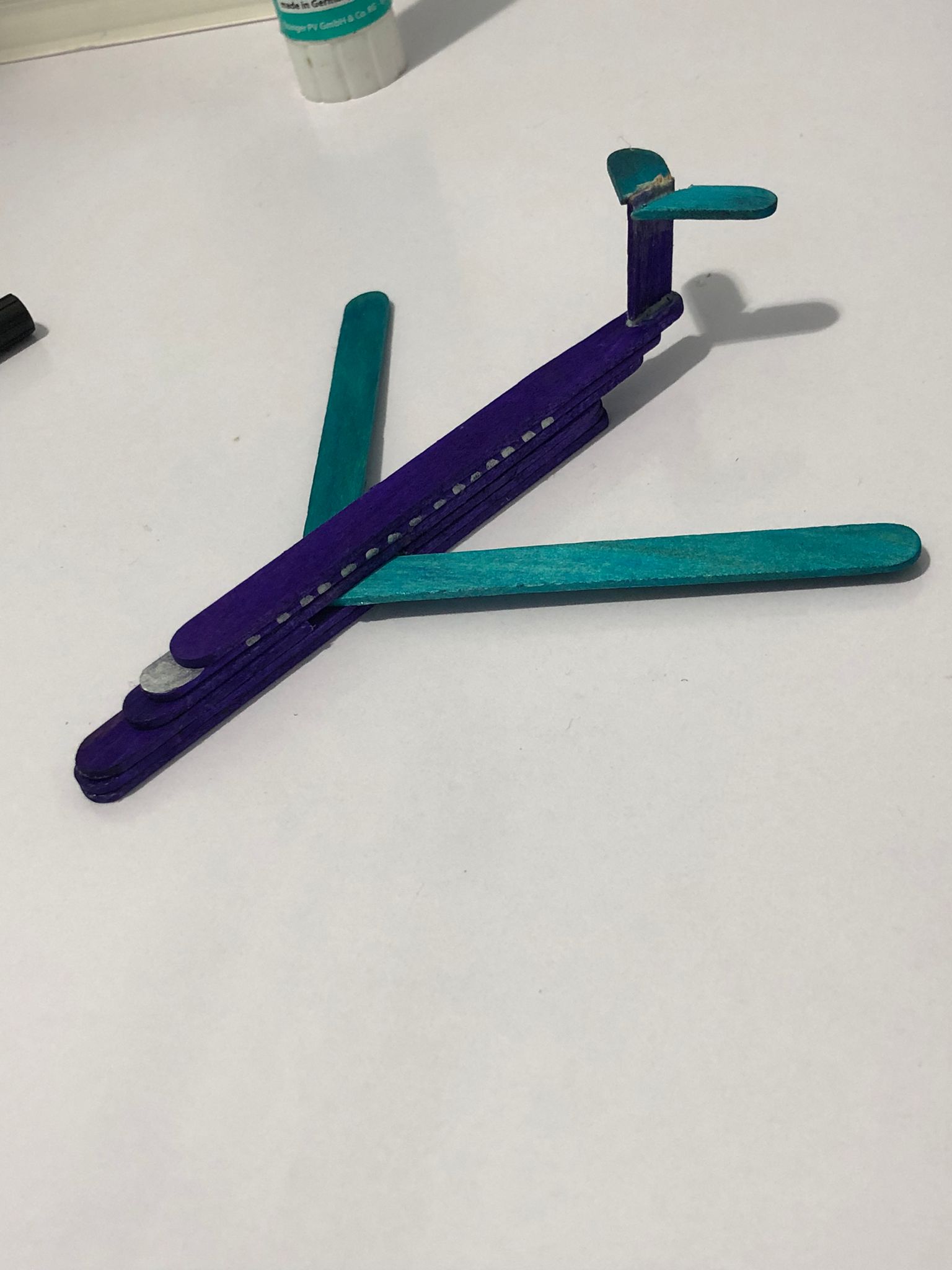


**Marble Maze**

The student designs and assembles a closed course on a rigid base through which a marble runs, which must reach the end without slipping out.

Improves movement control, spatial design and fine execution.

Materials: cardboard, scissors, glue, marbles.


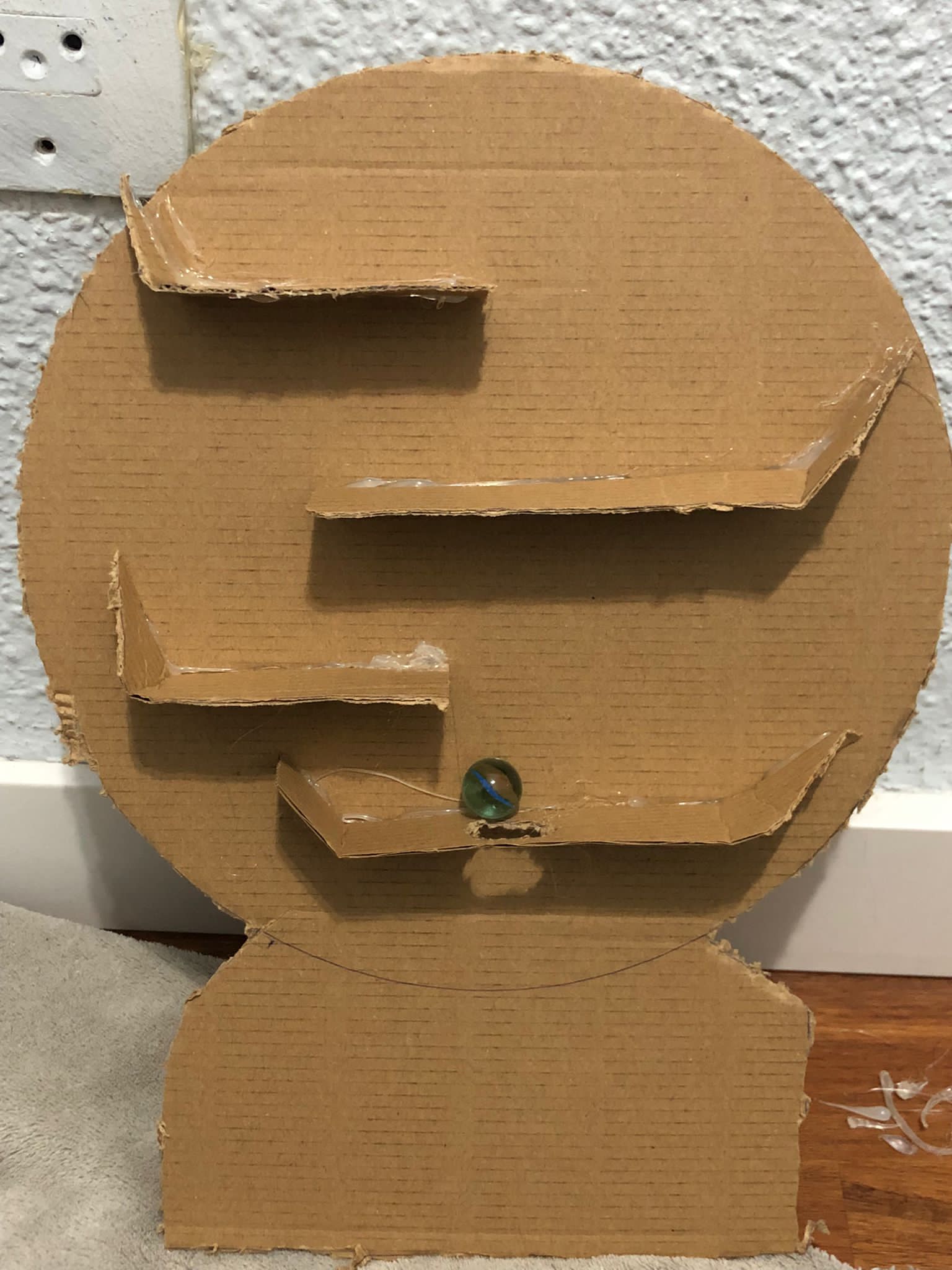


**Macramé dreamcatcher**

The student weaves a basic dreamcatcher using flat knot and lark's knot techniques. Work is done up to the middle of the central weave.

Encourages bilateral coordination, symmetry and thread tension control.

Materials: macramé thread, metal hoop, cardboard support.


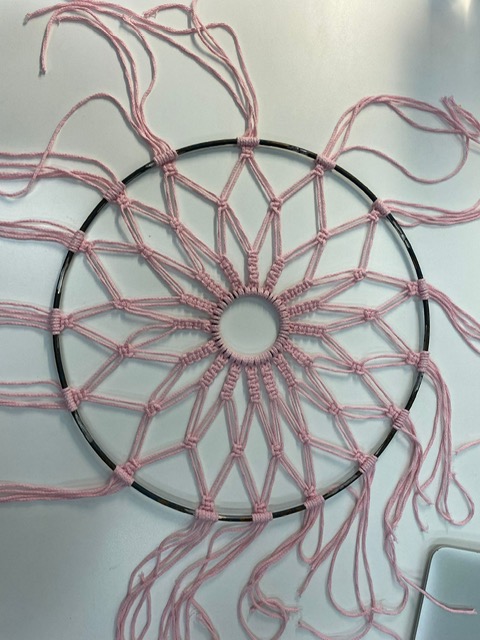


**Powered car**

A model car is made using recycled materials such as bottles, caps and rubber. The propulsion is generated by controlled elastic force.

It allows them to work on mechanical, design and motor control skills.

Materials: plastic bottle, caps, rubber bands, sticks.


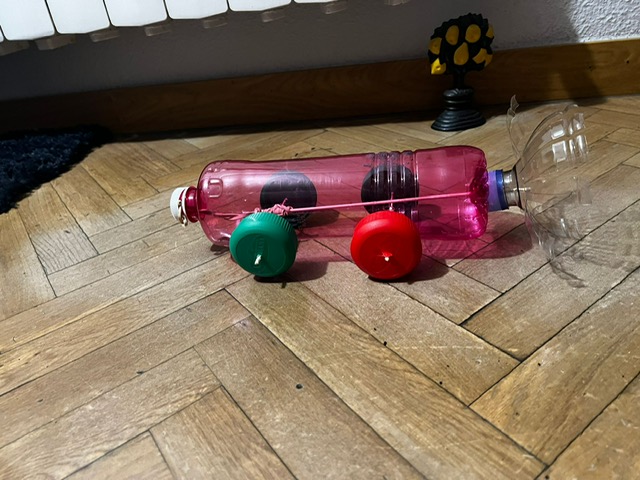


**Initial decorated with egg cups**

The activity consists of cutting and painting sections of egg cups simulating flowers, which are glued onto cardboard to form the initial of the pupil's name.

It promotes motor skills, precision and artistic composition.

Materials: egg cups, paint, cardboard, scissors, glue.


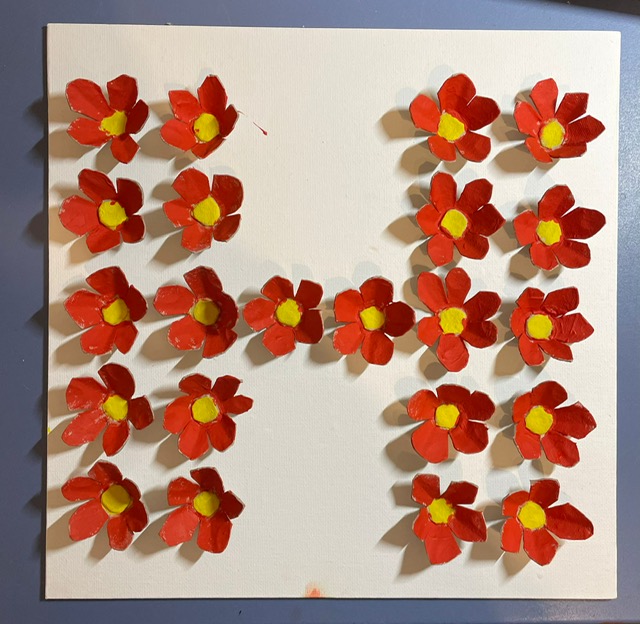


**Spoon and egg maze**

The student builds a cardboard path through which he/she must move a spoon holding an egg, preventing it from falling or touching the edges.

Requires fine control, balance and concentration.

Materials: spoon, egg (plastic or real), cardboard, scissors.


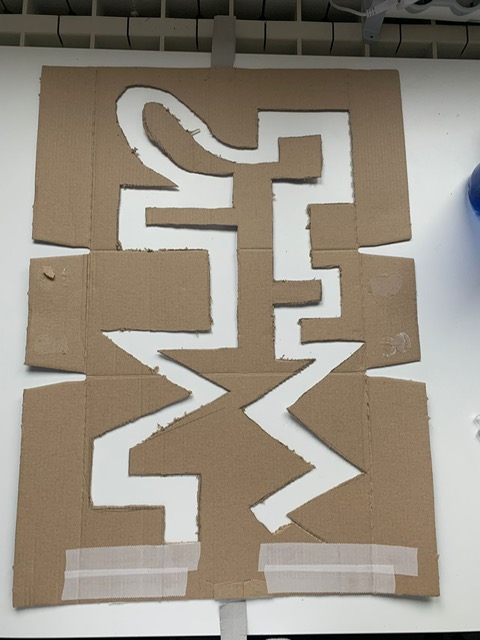


**Model boat**

A small boat is made with tongue depressors, plugs and complementary materials. Structural stability and functional design are sought.

Develops manual precision and problem solving.

Materials: tongue depressors, plugs, scissors, rubber bands, glue.


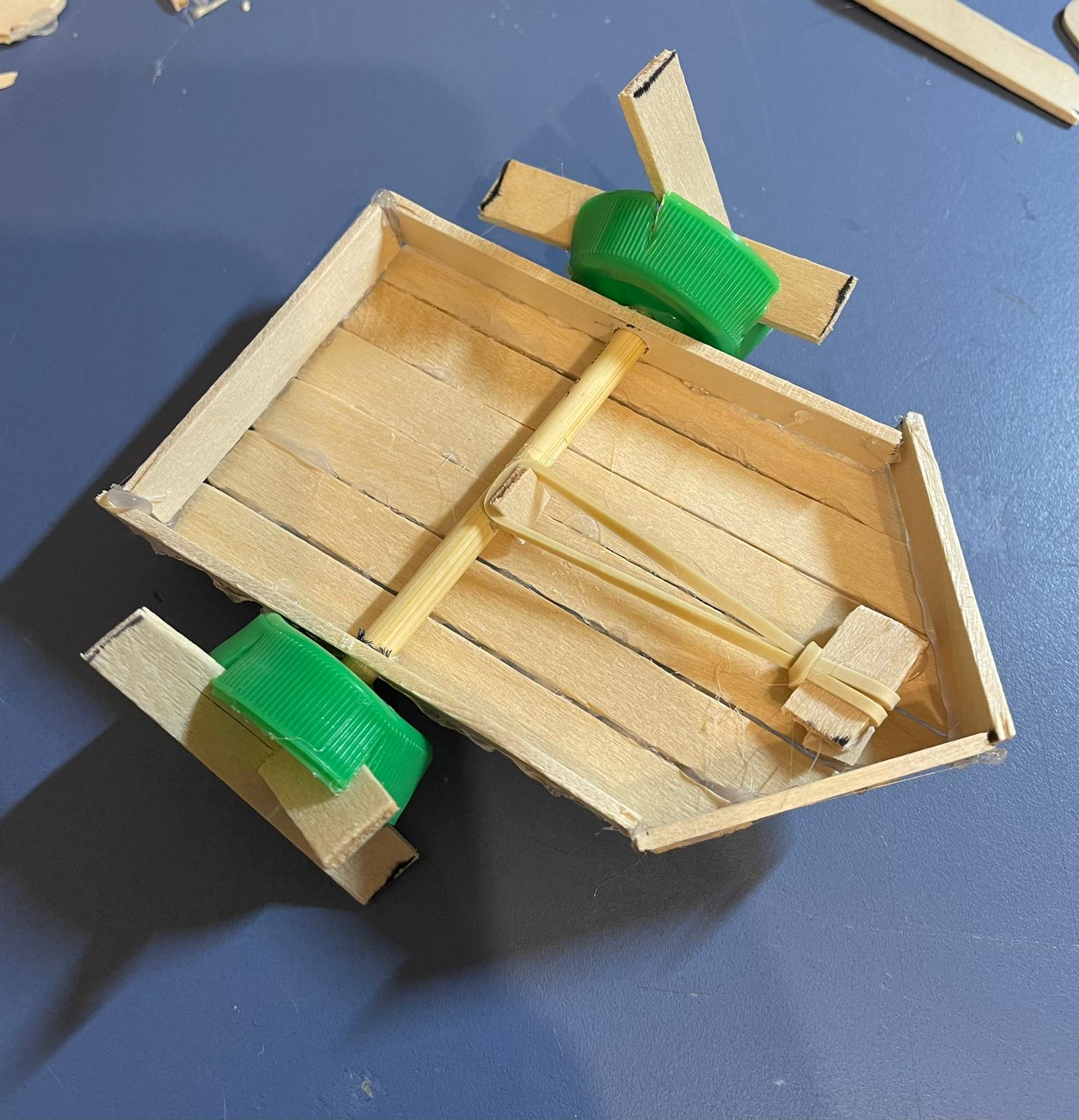


**Macramé leaf keyring**

Activity of repetitive leaf-shaped knots using macramé.

Requires symmetrical planning and repetition of motor sequences.

Materials: macramé thread, scissors, metal ring.


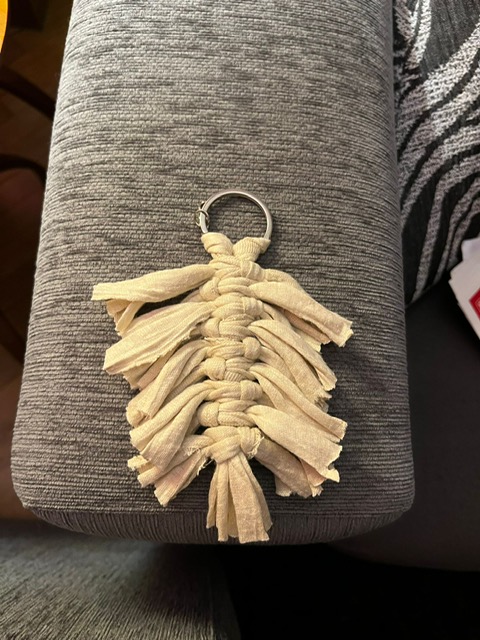


**Monthly Activities**

**Dance with balls**

Group choreography performed with balls, where body movement is synchronised with the musical rhythm. The final version is recorded after a long rehearsal.

Works on global coordination and teamwork.

Materials: balls, loudspeaker, large space.
